# Sensor Data Fusion Analysis for Broad Applications

**DOI:** 10.3390/s24123725

**Published:** 2024-06-07

**Authors:** Natividad Duro

**Affiliations:** Department of Computer Sciences and Automatic Control, Universidad Nacional de Educación a Distancia (UNED), C/Juan del Rosal, 16, 28040 Madrid, Spain; nduro@dia.uned.es

## 1. Introduction

Sensor data fusion analysis plays a pivotal role in a variety of fields by integrating data from multiple sensors to produce more accurate, reliable, and comprehensive information than that achieved by individual sensors alone. Advances in technology have allowed us to obtain relevant information from this analysis and many applications take advantage of this fact from different points of view. Not only can greater accuracy and reliability be achieved by combining data from multiple sources, but equally, better decision-making is executed, supported by informed decisions and predictive analytics from data fusion, which can be crucial for the operation of many industrial applications.

Therefore, efficiently integrating and analyzing data from multiple sources directly impacts the attainment of higher productivity and more efficient operations in industry. In this sense, resource optimization can be achieved by mitigating the need for additional sensors and reducing redundancy by leveraging data from existing sensors more effectively.

Different emerging areas gain relevant benefits from sensor data fusion analysis. These include industrial applications, medical or biomedical applications, robotics, monitoring systems, transportation systems, information systems, or control processes.

Applications in all of these areas require advanced algorithms and techniques to analyze and interpret the data from various sensors. To this end, it is still necessary to investigate several challenges such as reducing the complexity and computational load, especially in real-time applications, ensuring privacy and security, and maximizing data interoperability, among others.

## 2. Overview of Published Papers

All submissions were judged on technical merit and relevance, and sixteen high-quality papers were ultimately accepted to appear in this Special Issue.

Below, a list of accepted contributions is provided, each accompanied by a brief description. We believe that such papers offer updates and key insights into the research areas that this Special Issue focuses on and may therefore inspire future work in this area.

In the first contribution, the authors use RGB thermal fusion data as a robust neural network-based neonatal face detection method. They show that it is possible to achieve accurate sensor fusion for short distances using a ToF camera as an additional sensor. Its solution offers good precision, increasing data efficiency and economizing the process.

In the second paper, the authors present a novel methodology that provides an early fusion module capable of introducing the required reliability in a next-generation lightweight object detector in the event of sensor failure as well as extreme weather conditions. The article demonstrates that together, the early melt detector and the multimodal marker work robustly and transparently. Additionally, integrating a GPU enables the system to perform exceptionally well in real time.

The third study presents a new odometry system implemented in an autonomous wheelchair. It uses LSTM neural networks to estimate the speed of the robot using an encoder sensor. Real-time retraining allows the system to self-calibrate and adapt to changes in the defined model and also reduces the influence of some unsystematic errors. The paper shows that the proposed wheelchair localization is a robust solution for state estimation in challenging environments.

The fourth article addresses the great interest that the academic and scientific community has in the development of technologies related to geospatial and terrestrial phenomena. It reflects a strong trend of using the SVM method to evaluate large volumes of textual and geospatial data, as well as the use of Weka software. The results present the implementation of an innovative, practical, and systematic approach for information extraction and the recommendation of relevant knowledge. The method uses calibration parameters for each object and achieves results with high precision.

The fifth study implements a real-time PPC for MLS subject to dynamic uncertainty and external disturbances. A modified GFTSMM function has been developed based on the errors of the proposed PPC, so the tracking error variables quickly converge to the equilibrium point. Thanks to the designed observer, the approximate value of all uncertainty is known, which reduces vibration and improves control performance. The combination of GFTSMC, PPC, and MTOSMO is a novel strategy that guarantees a stable position of the controlled ball and the possibility of orbital tracking with good performance in real time.

The sixth article presents a solution in the field of health, safety, and fire to obtain temporally synchronous data from high-resolution sensors. The authors develop an energy-conserving multi-sensor fusion framework that powers low-power model-driven microcontrollers using machine learning. Likewise, they apply optimization techniques using anomaly detection modes to provide real-time information on demand that saves lives. The paper presents the application and results in a real-life healthcare scenario.

The seventh paper mainly focuses on designing a highly accurate trainable EKF-based localization framework using inertial measurement units for an autonomous ground vehicle with dead reckoning. The goal is to fuse it with a laser image for simultaneous detection and localization and for mapping estimation based on range sensors that improve performance. Convolution neural networks, backpropagation algorithms, and gradient descent methods are implemented in the system to optimize the parameters. Additionally, a unique cost function is used to train the models and improve the accuracy. The research is generic and applicable to various robot localization models assisted by inertial measurement units. 

Paper eight proposes an Echo State Network architecture that exploits sensor data fusion to detect failures in a scaled replica of an industrial plant. Thanks to their sparse weight structure, Echo State Networks function as recurrent neural network models, exhibiting low complexity and memory consumption, making them suitable for deployment on an Edge device. By analyzing vibration and current signals, the proposed model can correctly detect most of the faults that occur in the plant. The experimental results demonstrate the feasibility of the proposed approach and present a comparison with other approaches, where it is shown that the presented methodology is the best trade-off in terms of precision, recall, F1-score, and inference time.

The ninth study presents new data fusion approaches for the characterization of musts and wines based on biogenic amine and elemental composition. The paper applies inductively coupled plasma techniques to determine a wide range of elements. The authors obtain good descriptive models to describe the different compositions of the wines and musts using data fusion.

The tenth paper shows a multiblock approach to fuse processes and near-infrared sensors for online prediction of polymer properties. The main goal of the study is to explore the feasibility of multiblock regression methods to build real-time monitoring models that predict two quality properties of acrylonitrile butadiene styrene by fusing data from near-infrared and process sensors. Several prediction models taking advantage of sensor measurements have been created, which have provided good prediction results and allowed for the identification of the most relevant block data for the prediction of quality parameters. 

Paper eleven proposes an optimization framework for the large-scale field placement of optical sensors to improve border protection. Compared to the frequently used maximum area coverage approach, this method minimizes undetected passages in the monitored area. The paper takes into consideration both natural and built environmental coatings. The optimization is performed using a bacterial evolutionary algorithm. Therefore, the developed simulation framework based on ray tracing provides an excellent opportunity to optimize large areas.

In the twelfth study, different types of artificial neural networks are investigated to estimate the arrival time in acoustic emission signals. Convolutional neural network models and a novel capsule neural network are proposed. The models have been tested with experimental data acquired in the framework of a localization problem to identify targets with known coordinates on a square aluminum plate. The models have been shown to have excessive precision at significant noise levels.

Paper thirteen evaluates the performances of five clustering algorithms: k-means, fuzzy C-means (FCM), hierarchical, mean shift, and density-based spatial clustering of applications with density-based noise. The paper analyzes the impacts of input data format and feature selection on the quality of management zone delineation. It uses key soil fertility attributes collected with an online visible and near-infrared spectrometer, demonstrating that k-means is the optimal clustering method for management zone delineation.

Paper fourteen proposes the use of the Delicar system, a self-driving product delivery vehicle that can drive the vehicle on the road and report the current geographical location to the authority in real time through a map. A camera captures the road image and transfers it to the computer using socket server programming. The system’s infrastructure is also low-cost and easy to install.

The fifteenth paper proposes a recent iterative learning algorithm for sensor data fusion to detect pitch actuator failures in wind turbines. The development of this proposed approach is based on iterative learning control and Lyapunov’s theories. Numerical experiments have been carried out to support the study. These experiments use a well-known model of a wind turbine hydraulic pitch actuator with some common faults, such as high oil content in the air, hydraulic leaks, and pump wear.

The last paper presents a detailed review of state-of-the-art data fusion solutions for data storage and indexing from various types of sensors, feature engineering, and multimodal data integration. The review aims to serve as a guide for the early stages of an analytic pipeline of manufacturing prognosis. The reviewed literature showed that in fusion and preprocessing, the methods chosen to be applied in this sector are beyond the state of the art. Existing weaknesses and gaps that will lead to future research were also identified.

## 3. Conclusions

In summary, the sixteen papers collected in this Special Issue represent a good example of the uses of data sensor fusion in industrial applications. The papers show a wide interest in the research area. 

Future research on sensor data fusion analysis will be focused on the miniaturization of sensors and components, as well as an increased use of multi-sensor systems and wireless and autonomous radio sensors. Developing smaller and smaller sensors will allow us to understand the world as we know it more easily and accurately. On the other hand, one of the main challenges in sensor fusion using mobile devices will be the quality of the data collected. Mobile sensors are easily affected by noise, interference, calibration errors, and outliers.

